# Maternal education and childhood immunization in Turkey

**DOI:** 10.1002/hec.3770

**Published:** 2018-05-22

**Authors:** Mustafa Özer, Jan Fidrmuc, Mehmet Ali Eryurt

**Affiliations:** ^1^ Faculty of Economics and Administrative Science Kilis Yedi Aralık University Kilis Turkey; ^2^ Economics and Finance Department University of Portsmouth Portsmouth UK; ^3^ Department of Economics and Finance and CEDI Brunel University Uxbridge UK; ^4^ University of Social and Administrative Affairs Havířov Czech Republic; ^5^ Institute for Strategy and Analysis (ISA) Government Office of the Slovak Republic; ^6^ CESifo Munich Germany; ^7^ Institute of Population Studies Hacettepe University Ankara Turkey

**Keywords:** difference‐in‐difference‐in‐difference, diphtheria, pertussis, and tetanus (DPT), Hepatitis B, instrumental variable, maternal education, vaccination

## Abstract

We study the causal effect of maternal education on childhood immunization rates. We use the Compulsory Education Law of 1997, and the differentiation in its implementation across regions, as instruments for schooling of young mothers in Turkey. The Compulsory Education Law increased the compulsory years of schooling of those born after 1986 from 5 to 8 years. We find that education of mothers increases the probability of completing the full course of diphtheria, pertussis, and tetanus and Hepatitis B vaccinations for their children. The results are robust to variations in regression specification and including various individual and community variables.

## 1 INTRODUCTION

According to a report by the World Health Organization and the United Nations Children's Funds, some 8 million children under five die annually, and 17% of those deaths could have been prevented if the children were vaccinated. 1World Health Organization and United Nations Children's Funds: Global Immunization Data; July 2014. Accessible at http://www.who.int/immunization/monitoring_surveillance/global_immunization_data.pdf (cited on 24/07/2015). The same report estimates that 1–3 million additional deaths from measles, diphtheria, pertussis (whooping cough), and tetanus (DPT) are prevented by vaccination each year. The potential gains from increased vaccination coverage are particularly large in less developed countries, where vaccination rates remain low, especially in rural areas. In Turkey, the percentage of children who are fully vaccinated increased from 46% in 1998 to 81% in 2008 (Table 1). 2Children who are fully vaccinated are those who have received vaccinations against tuberculosis (Bacillus Calmette–Guérin [BCG]), measles, and three doses of DPT, Hepatitis B, and polio.


**Table 1:** Trend of full vaccination in Turkey

Mother's education	1998	2003	2008
No education/primary incomplete	28.5	26.1	64.9
Primary school	48.0	60.9	81.6
Lower secondary school	64.0	61.2	84.4
Higher school		68.5	87.8
Total	45.7	54.2	80.5

Source: Author's own calculation based on TDHS‐1998, TDHS‐2003, and TDHS‐2008.

The likelihood that a child will be vaccinated, however, is not random. Rather, it appears closely correlated with the mother's level of education. The relationship between maternal education and children's immunization rates has been highlighted also in the previous literature (Abuya, Onsomu, Kimani, & Moore, 2011; Altınkaynak, Ertekin, Güraksın, & Kılıc, 2004; Fatiregun & Okoro, 2012; Schoeps et al., 2013; Singh, Haney, & Olorunsaiye, 2013; Vikram, Vanneman, & Desai, 2012). However, this relationship is not necessarily causal, for a number of reasons. First, both maternal education and vaccination take‐up might be driven by (household) income (Behrman & Rosenzweig, 2002). Second, the relationship between education and any outcome of interest may be distorted by the “ability bias” (Card, 1999; Griliches, 1977). Third, a reverse causality problem might also exist between education and fertility choices because a woman's education is likely to be affected by the timing of child bearing (Angrist & Evans, 1998; Jensen & Thornton, 2003). The instrumental variable (IV) method can be used to overcome these problems.

A number of papers seek to control for the potential endogeneity of education using natural experiments, whereby the exposure to an education reform by date of birth and the differentiation in its implementation across regions serve as an instrument for schooling of mothers. Breierova and Duflo (2004) in Indonesia, Chou, Liu, Grossman, and Joyce (2010) in Taiwan, and Osili and Long (2008) in Nigeria find a negative correlation between maternal education and fertility and child mortality. In the context of Turkey, Güneş (2015) exploits the variation in the number of classrooms constructed across regions, whereas Dinçer, Kaushal, and Grossman (2014) use the variation in the number of teachers recruited after the change in compulsory education in Turkey in 1997 as a measure of the reform intensity. Cesur, Dursun, and Mocan (2014) investigate the causal impact of education on the propensity to get flu shot for adults. All three studies use exposure to the 1997 compulsory education reform (discussed below) to tease out the effect of education on the outcome of interest. Güneş (2015) and Dinçer et al. (2014) conclude that maternal education improves children's mortality, birth weight, and height and weight for age. In contrast, Cesur et al. (2014) find no impact of education on the probability of getting the flu vaccination or on other health‐related behaviors (of adults).

The present paper, in contrast, considers the effect of adults' (and specifically mothers') education on children's rather than adults' own vaccination rates. To the best of our knowledge, our paper is the first study to explore the relationship between maternal education and children's vaccination rates in an analytical setting that takes due account of the likely endogeneity bias. We select Turkey for a number of reasons. First, it is a developing country with less than perfect immunization coverage. Therefore, findings from Turkey will be of relevance to other countries at similar level of development and with similarly patchy immunization records. Second, we can use a unique comprehensive data set for Turkey, the Turkey Demographic and Health Survey (TDHS), which includes not only information on maternal education and childhood immunization but also a broad range of regional and individual level variables. Third, Turkey offers a unique natural experiments allowing us to address the likely endogeneity bias of education. Specifically, Turkey expanded compulsory education from 5 to 8 years in August 1997. This extension in compulsory education in turn generated an urgent need for the construction of new classrooms and employment of new teachers, which necessitated an increase in the government spending on education. The increase in government expenditure was not distributed equally across the country. We can therefore compare the age cohorts on either side of the cutoff age for the reform 3This reform affected children aged 10 or less in the 1997–1998 school year. and also exploit the differences in the additional spending for classroom construction across regions in an IV framework to estimate the effect of maternal schooling on children's vaccination rates.

Given that the compulsory education reform took place in 1997, our analysis is restricted to young mothers. Specifically, in the 2008 wave of the survey, women aged 21 and younger were required to stay in school for at least 8 years; women aged 22 and above were subject to 5 years of compulsory education. We, therefore, restrict our attention to women aged 18–21 (*treatment group*) and 22–29 (*control group*) in 2008. We use also an earlier wave of the survey, from 2003, to tease out the effect of age on children's immunization. Neither the young nor old women in that survey were affected by the reform. Rather, using both waves allows us to control for the fact that young and old mothers may have different compliance rates with vaccination schedules.

Our findings suggest that maternal education, measured both by years of education and as completion of 8 years of education, significantly improves the take‐up of the last doses of Hepatitis B and DPT immunizations. These findings are very robust to alterations in the regression specifications and to accounting for individual and community level variables. These results suggest that education has not only labor‐market returns but is also associated with important nonmarket benefits, including those that affect the next generation.

The next section outlines the data used for this study. Section 3 explains our empirical strategy. Section 4 presents the findings of our research. Finally, section 5 gives a summary of our findings.

## 2 DATA AND EMPIRICAL FRAMEWORK

Our study combines the last two rounds of the TDHS conducted in 2003 and 2008. The surveys were designed to capture trends and levels of fertility, infant and child mortality, family planning, and maternal and child health including preventive health measures (e.g., the childhood vaccination status) of ever‐married women. The TDHS surveys also include a wide range of information on women's socioeconomic characteristics, such as education (completed years of schooling and the highest level of education attained), parents' education, employment status, ethnicity, women's status in the family, and demographic information including age, gender, type of birth place (rural/urban), the region of birth, and the region of residence during childhood. Note that childbearing outside of marriage is uncommon in Turkey, so that by only considering married women, we are unlikely to omit any significant number of mothers. Our final sample consists of 3,331–3,382 young mothers between the ages 18 and 29 in both the 2003 and 2008 TDHS. Recall that the women aged 18–21 in 2008 were affected by the reform, whereas the older women in the same wave were not affected. None of the women interviewed in 2003 were affected; they are included in our analysis so as to distinguish the effect of the exposure to compulsory education reform from that of becoming a mother at a slightly older age. 4The downside of considering young mothers only is that we may fail to capture the full effect of education by missing the women who postpone childbearing until after completion of their tertiary education. However, it is unlikely that the education reform has had a dramatic impact on these women as most of them probably would have continued in their education until university even without the reform. The literature offers contradictory findings in this respect. On the one hand, Kirdar, Tayfur, and Koç (2011) find that while CEL reduced childbearing and marriage for women aged 17 and less, no effect was observed for women aged 18 and over. On the other hand, Ergöçmen (2012) finds that Turkish women tend to delay marriage and first childbirth over time. If this delay is due to educational attainments rising over time, this can lead to a bias undermining our results.


Two dichotomous variables are used to measure the completion of immunization for children aged over 6 months: DPT and Hepatitis B vaccines. Each takes the value of one if the child has received the third and final dose. 5We include these two vaccines as both require three doses for full protection against these diseases: The take‐up of the second and third doses tends to be lower than for the first dose so that there is more variation across mothers with respect to these two vaccines compared to others. The third dose is to be received by the time the child is 6 months old: We, therefore, only include mothers of children older than 6 months (specifically, the children are aged 6–23 months in 2003 and 6–26 months in 2008). We do not consider polio or BCG. During the period covered by the two surveys, Turkey was a polio‐free country following a series of mass national immunization campaigns against this disease repeated every year since 1997. As for BCG, this vaccine is administered to infants only once, unlike DPT or Hepatitis B. We also construct two education variables: years of education as a continuous variable and a dummy variable capturing whether the woman completed 8 years of schooling (junior high school [JHS]). 6The women in our sample no longer remain in education. This means that the educational data obtained from TDHS represent the final education level of women.


To control for the unobserved time‐invariant effects of the childhood environment (such as disparities in socioeconomic development among regions, differences in school and teacher quality, and their availability in the prereform period) on schooling outcomes, we include dummy variables for 26 regions in which the women spent their childhood until the age of 12. 7The Turkish Statistical Institute divides Turkey into 26 subregions at Statistical Regional Classification Unit level (or Nuts‐2 level; see Turkish Statistical Institute website: http://www.turkstat.gov.tr/Start.do). The women's region of residence until the age of 12 allows us to identify the impact of compulsory schooling reform on education of women, as it allows us to link individual survey data with regional administrative data. Furthermore, we control for rural/urban type of birth place, ethnicity and include fixed effects for the mother's year of birth (the latter serves to account for the potential impact of government policies, as well as changes in the utilization of healthcare services and education preferences across cohorts). We also include dummies for the baby's birth order to account for the fixed effects of the mother's previous birth experiences about vaccination, and a dummy representing the child's gender.

A problem may arise if the regional intensity of public spending on classroom construction is higher in regions with lower prereform enrolment rates in Grades 6–8. To deal with this issue, we add an interaction of the year of birth fixed effect with the gross enrolment rate in 1996–1997 in the childhood region prior to education reform. 8Gross enrolment rate in JHS, that is, Grades 6–8, is calculated by dividing the number of children who are enrolled in JHS in 1996 in the childhood region of children by the population of children aged 11–13 in the same region and year. The number of JHS students was obtained from the Ministry of National Education's National Education Statistics. The school‐aged population in 1996 was based on the censuses conducted by the Turkish Statistical Institute in 1990 and 2000. This controls for the potential link between the intensity of the implementation of the education reform, and the enrolment rates before the reform and other unobservable factors related to these enrolment rates.

## 3 EMPIRICAL STRATEGY

The December 1995 election in Turkey resulted in the victory of a religious Welfare Party. On June 28, 1996, the Welfare Party formed the first government in Turkey's modern history that was led by an Islamist party. On February 28, 1997, the National Security Council, dominated by the military, forced the government to resign as its religious orientation was seen as a threat to democracy and secularism. The same meeting of the National Security Council decided to increase compulsory education from 5 to 8 years, which was implemented by the Turkish Parliament in August 1997. The stated objective of the reform was to ensure that children across all of Turkey would receive education of adequate and uniform standard for the first 8 years of their schooling (Dulger, 2004a, 2004b). This included bringing all students in Grades 1–8 into public schools under a national curriculum, thus, eliminating the previously available options of private, foreign, or religious education. Religious education, especially, was popular prior to the reform: By 1997, 11% of children enrolled in postprimary education were attending religious schools (Buyruk, 2015; Cornell, 2015). The reform also made it almost impossible for graduates of religious secondary schools (so‐called imam and preacher schools) to gain entry to universities to study subjects other than theology. Consequently, following the reform, the share of students in religious schools fell precipitously, to 2% (Cornell, 2015). An additional motivation for the reform was the ambition to secure Turkey's entry into the EU, and increasing the level of education was seen as an important step in that direction (Cesur et al., 2014; Dulger, 2004a, 2004b). Importantly, both the desire to restrict religious education and the intention to support Turkey's EU accession were political motivations uncorrelated with the educational or health outcomes in Turkey immediately before the reform. This allows us to use the reform as a natural experiment constituting an exogenous instrument for educational attainments.

**Figure 1:**
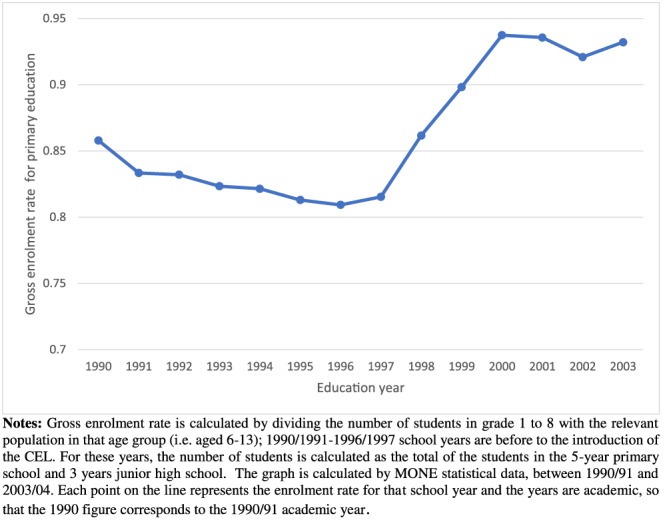
The trend of gross enrolment rate in 8‐year primary schools in Turkey. Notes: Gross enrolment rate is calculated by dividing the number of students in Grades 1–8 with the relevant population in that age group (i.e., aged 6–13); 1990/1991–1996/1997 school years are before to the introduction of the CEL. For these years, the number of students is calculated as the total of the students in the 5‐year primary school and 3 years junior high school. The graph is calculated by MONE statistical data, between 1990/1991 and 2003/2004. Each point on the line represents the enrolment rate for that school year and the years are academic, so that the 1990 figure corresponds to the 1990/1991 academic year [Colour figure can be viewed at http://wileyonlinelibrary.com]

Figure 1 demonstrates that there was a decreasing trend in gross enrolment rates prior to the reform. The reform led to a substantial increase in overall gross enrolment. To meet the additional demand for school places, 81,500 new primary‐school classrooms were constructed between 1997 and 2002, which corresponds to an almost 40% capacity increase (World Bank, 2005), and 70,000 new teachers were employed (Dulger, 2004a). This necessitated increasing the budget for primary school construction by 30% from 1996–1997 to 1997–1998 school years. 9Source: Statistical Yearbook on Public Expenditure from 1996–1997 to 1997–1998 education year, Turkish Ministry of Development. The first cohort affected by this change were the children who started the fifth grade in the 1997–1998 academic year. Because school enrolment in Turkey is determined according to calendar years rather than school years, 10The law states that “child who has completed 72 months by the end of the calendar year can be registered to the first degree of primary school,” according to the law published in edition No. 21308 of the official newspaper of the Republic of Turkey on Friday, August 7, 1992. children born in or after 1987 (aged 10 or less in 1997–1998) were affected by the education reform whereas those aged 11 or more were not. 11However, the implementation of the age cutoff was not strict: Some children born in early 1986 might start school in September 1991 instead of September 1992, whereas some other children might start school in September 1993. This means that some pupils born in 1986 could have been affected by the education reform, potentially contaminating the results. Therefore, the 1986 cohort was excluded from the estimation as a robustness check. This, however, yielded results which were not materially different from those presented below.


In this paper, we use the 3‐year exogenous change in educational attainment triggered by the timing of the education reform as an instrument for education. One of the requirements of a valid instrument is that it should not have any impact on the outcome variable other than its influence through schooling. We believe that the education law reform meets this condition. First, the compulsory schooling reform was instigated by the political events in 1997, so that it has no link to the outcome variables. Second, the factors typically blamed for causing endogeneity of education, such as the innate ability and other individual characteristics, are not likely to be linked to the year of birth.

**Figure 2:**
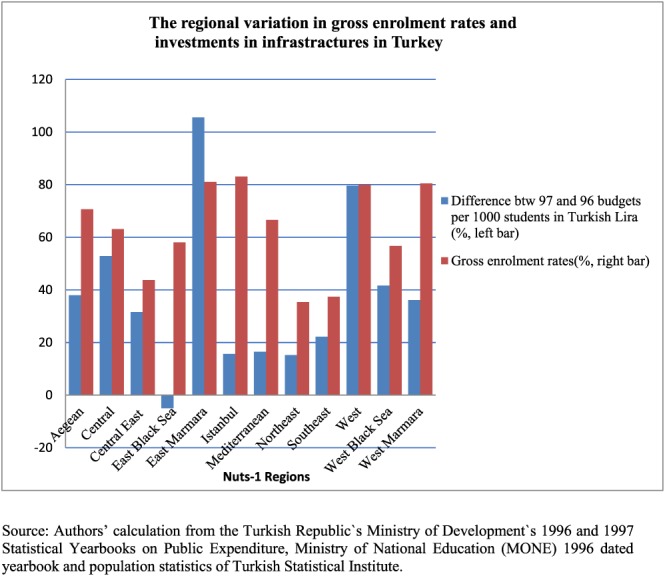
The regional variation in gross enrolment rates and investments in infrastractures in Turkey. Source: Authors' calculation from the Turkish Ministry of Development's 1996 and 1997 Statistical Yearbooks on Public Expenditure, Ministry of National Education (MONE) 1996 dated yearbook and population statistics of Turkish Statistical Institute [Colour figure can be viewed at http://wileyonlinelibrary.com]

Relying solely on the variation in the birth years as an instrument for schooling might lead to a bias in the estimations because there might be other unobserved events taking place at the same time as the education reform. Therefore, we also employ the intensity of the education reform, measured as the difference in additional expenditure on classroom construction per 1,000 children between 1997–1998 and 1996–1997 education years in the childhood region of the mother. 12The public expenditure figures are based on information from the Turkish Ministry of Development's 1996 and 1997 statistics yearbooks and are adjusted for inflation. The Turkish Statistical Institute's 1990 and 2000 census statistics were used to estimate the population aged 6–13 in 1996 and 1997, with missing data estimated using the exponential function method. The estimations were made also using the difference between 1998 and 1996 as a robustness check. The change in the measure of intensity does not have an impact on the outcomes of interests. Because the identification assumption requires that individuals affected by the education reform experienced a higher intensity of construction expenditures, the intensity is required to be conditionally random. At first glance, the condition for the identification assumption seems to be satisfied. Figure 2 shows that there is little correlation between the enrolment rates in 1996 education year and the additional expenditures on classroom constructions: The allocation of additional funds for classroom construction appears as good as random, making it, in combination with the year of birth, a good measure of the reform impact and therefore a good instrument for schooling (see Duflo, 2001). 13In addition, in order to eliminate the potential bias, we account for the potential unobserved time‐invariant impact of childhood environment on the distribution of additional spending on classroom construction across regions by controlling for the childhood region of residence and rural/urban characteristic. We also include the interaction of year‐of‐birth fixed effect with the gross enrolment rate in the childhood region in 1996–1997 school year, just prior to the education reform. This accounts for the differentiation in the intensity of the compulsory education reform correlated with the enrolment rates before the reform at JHS (Grade 6–8) and other unobservable factors related to these enrolment rates across cohorts.


Women born between 1987 and 1990, who were affected by the education reform, therefore form the treatment group, and those born between 1979 and 1986 are in the control group. The schooling decision of the individuals could be estimated with the standard difference‐in‐difference methodology, utilizing the 2008 survey, as follows 14This model was constructed similarly as in Duflo (2001).:

(1) $$S_{\mathrm{ijt}}=\mu^{S}+\beta_{l}^{S}+\gamma_{j}^{S}+\theta_{t}^{S}\left({\text{young}}_{i}*{\text{intensity}}_{j}\right)+\theta^{S}{\text{intensity}}_{j}+X_{\mathrm{ijt}}\pi^{S}+\varepsilon_{\mathrm{ijt}}^{S},$$

where *S*_*ijt*_ denotes the educational attainment of mother *i* who lived in the childhood region *j* and was interviewed for the TDHS survey in year *t*. As indicated previously, there are two education variables, years of education and a dummy for completing 8 years of formal education. The *young*_*i*_ variable equals 1 for the treatment group and 0 for the control group, whereas *intensity*_*j*_ captures the regional variation in the intensity of education reform in the childhood region of women. $\beta_{l}^{S}$ indicates the year of birth fixed effect; *γ*_*j*_ is the fixed effect for the region in which the woman lived most of her life until age 12; and, finally, the remaining control variables are represented by *X*_*ijt*_. These are ethnicity, the interaction of year of birth with gross enrolment rate in 1996–1997, and dummies indicating the birth order of the baby and the gender of the baby.

The correlation between schooling and the reform for the treatment group is estimated by $\theta_{t}^{S}+\theta^{S}$ whereas the same relationship for the control group is captured by *θ*^*S*^ Therefore, $\theta_{t}^{S}$ captures the impact of the compulsory education reform on the formal schooling of the treatment group, if the control and treatment groups are equally influenced by the other determinants associated with the intensity variable and the reform was exogenous.

Up to now, the discussion has focused on the assumption that the exposure of women to the Compulsory Education Law (CEL; i.e., the instrument) is jointly determined by the year of birth and region of childhood. This assumption implies that factors related to the intensity of public investment on classroom construction have identical influence on mothers in the treatment and control groups. However, if mothers' outcomes such as their use of preventive health measures for their children (e.g., vaccination) vary by age, any method that does not compare treated and untreated women of the same age would be biased. The difference‐in‐difference methodology, however, cannot account for the impact of age on the outcome of interest when treatment is determined by age. For that reason, we use the difference‐in‐difference‐in‐difference (DDD) strategy. This methodology assumes that the education choices of individuals are a function of the date of birth, additional government spending on classroom construction in the region of childhood, and age. It does so by controlling for both year of birth fixed effects and age fixed effects whereas difference‐in‐difference methodology only controls for the former (see Dinçer et al., 2014). In order to implement the DDD method, 2003 and 2008 waves of TDHS are pooled for mothers aged 18 to 29 in both surveys. These are again divided into two subgroups: young (aged 18–21) and old (aged 22–29). As a result, the DDD methodology can be applied in this setting with the combination of 2003 and 2008 TDHS as follows:

(2) $$S_{\mathrm{ijt}}=\propto^{S}+\omega_{l}^{S}+\beta_{j}^{S}+\beta_{a}^{S}+\theta_{t}^{S}\left({\text{young}}_{i}*{\text{intensity}}_{j}*2008\right)+\theta_{y}^{S}\left({\text{young}}_{i}*{\text{intensity}}_{j}\right)+\theta^{S}{\text{intensity}}_{j}+X_{\mathrm{ijt}}\pi +\varepsilon_{\mathrm{ijt}}^{S}.$$

In the above regression, $\beta_{a}^{S}$ stands for the age fixed effect; $\omega_{l}^{S}$is the year of birth fixed effect; $\beta_{j}^{S}$ is the region of childhood fixed effect; and 2008 is a dummy for the TDHS 2008 cross section. The year of birth fixed effect controls for general trends in the outcome of interest caused by other changes specific to birth cohorts. Controlling for the birth year ensures that mothers between the ages of 18 and 21 in TDHS 2003 and 2008 have identical trends related to educational attainment, utilization of immunization services for their babies, and potential mechanisms affecting vaccination usage in the absence of reform. $\theta_{y}^{S}$ measures how the impact of the intensity of public spending on classroom construction varies between the young (aged 18–21) and old (aged 22–29) women who participated in the 2003 wave of the survey. Finally, $\theta_{t}^{S}$ captures the impact of the reform intensity on the education of young mothers aged 18 to 21 who participated in the TDHS 2008.

The correlation between maternal education and childhood immunization in an Ordinary Least Squares (OLS) setting could be estimated as follows:

(3) $$Y_{\mathrm{ijt}}=\propto^{Y}+\omega_{l}^{Y}+\beta_{j}^{Y}+\beta_{a}^{Y}+\delta^{Y}S_{\mathrm{ijt}}+\delta_{y}^{Y}\left({\text{young}}_{i}*{\text{intensity}}_{j}\right)+\theta^{Y}{\text{intensity}}_{j}+X_{\mathrm{ijt}}\pi^{Y}+\varepsilon_{\mathrm{ijt}}^{Y},$$

where *Y*_*ijt*_ is a dummy variable which equals 1 if children were vaccinated against the third (last) dose DPT or Hepatitis B and 0 otherwise. OLS estimates of *δ*^*Y*^ might be biased because it is possible that schooling is correlated with the error term. On the other hand, if the reform only affects the outcome of interest through education, that is, the reform has no direct effect on the dependent variable (vaccination rates), then, the results of DDD estimates in Equation (2) capture the effects of the CEL on maternal education. The triple interaction term *young*_*i*_ * *intensity*_*j*_ * 2008 from Equation (2) then can be used as the instrument for the schooling of mothers, so as to obtain unbiased estimates of the effect of education on the outcome considered.

Our IV model, therefore, is as follows:

(4) $$Y_{\mathrm{ijt}}=\propto^{Y}+\omega_{l}^{Y}+\beta_{j}^{Y}+\beta_{a}^{Y}+\delta^{Y}\hat{S_{\mathrm{ijt}}}+\delta_{y}^{Y}\left({\text{young}}_{i}*\text{inte}n{\text{sity}}_{j}\right)+\theta^{Y}{\text{intensity}}_{j}+X_{\mathrm{ijt}}\pi^{Y}+\varepsilon_{\mathrm{ijt}}^{Y}.$$

Note that education was replaced by $\hat{S_{\mathrm{ijt}}}$, the predicted value of education. It is also important to note that instead of IV‐Probit or Logit, we use conventional 2SLS estimation technique as suggested by Angrist (1991) and Angrist (2001) because the dependent and endogenous variables, as well as the instrument, are dichotomous. Under this condition, estimates identify the marginal treatment effect irrespective of whether the dependent variable is binary or continuous and are more robust than estimates obtained with nonlinear models (Angrist & Pischke, 2009, pp. 197–198).

Finally, a modification of Equation (2) (the first stage regression) yields reduced form (RF) estimates where the education outcome of interest in the first stage is replaced with vaccination status as follows:

(5) $$Y_{\mathrm{ijt}}=\propto^{Y}+\omega_{l}^{Y}+\beta_{j}^{Y}+\beta_{a}^{Y}+\theta_{t}^{Y}\left({\text{young}}_{i}*{\text{intensity}}_{j}*2008\right)+\theta_{y}^{Y}\left({\text{young}}_{i}*{\text{intensity}}_{j}\right)+\theta^{Y}{\text{intensity}}_{j}+X_{\mathrm{ijt}}\pi^{Y}+\varepsilon_{\mathrm{ij}t}^{Y}.$$

In all regressions that we estimate, standard errors are clustered for the 26 regions of childhood. A problem might arise if the number of clusters is less than around 42–50, so that the null hypothesis may be rejected even when it is true (Angrist & Pischke, 2009; Bertrand, Duflo, & Mullainathan, 2004). However, Cameron, Gelbach, and Miller (2008) argue that the null hypothesis is less likely to be rejected when it is true if the number of clusters is around 20 than when it is 50. Therefore, it is likely that the number of cluster is enough to obtain reliable estimates.

Table 2 presents descriptive statistics for the old and young cohorts in both surveys. Several notable observations can be made. In both surveys, young mothers marry and give birth for the first time at considerably younger age: This is not surprising, given the construction of the two groups. In 2003, when neither old nor young mother were affected by the education reform, old mothers have almost 1 year more of schooling on average and are also more likely to complete 8 years of school (JHS). In 2008, this pattern reverses and the young cohort has slightly more education and is almost twice as likely to have completed the JHS as the old cohort. A similar reversal occurs with respect to vaccination rates: In 2003, old mothers are significantly more likely to have their children fully vaccinated while the difference between the two groups disappears by 2008. Reassuringly, we see very little difference with respect to the other variables, especially those that should be independent of age or exposure to educational reform: rural versus urban origin or ethnicity. In the next section, we explore whether there is indeed a causal relationship between the observed increase in education among the young mothers in 2008 and the improvement in vaccination rates of their children compared to young mothers in 2003.

**Table 2:** Descriptive statistics

	TDHS 2003				TDHS 2008			
	Old		Young		Old		Young	
	aged 22–29		aged 18–21		aged 22–29		aged 18–21	
Description of selected variables	Obs	Mean	Obs	Mean	Obs	Mean	Obs	Mean
Variables								
Children immunized against DPT3	1918	0.624	316	0.520	952	0.821	194	0.810
Children immunized against Hepatitis3	1918	0.491	316	0.362	953	0.788	194	0.772
Age at first marriage	1918	18.956	316	16.471	954	19.680	194	16.641
Age at first birth	1918	22.968	316	18.329	954	23.960	194	18.345
Years of schooling	1918	5.831	316	4.906	954	6.146	194	6.384
Completing 8 years of schooling	1918	0.276	316	0.173	954	0.298	194	0.571
Ethnicity	1918		316		954		194	
Turkish		0.746		0.672		0.738		0.736
Kurdish		0.216		0.290		0.231		0.248
Other		0.038		0.038		0.031		0.027
Rural/urban status during childhood	1883		312		944		193	
Rural		0.510		0.528		0.451		0.400
Urban		0.490		0.472		0.549		0.600
Child‐gender dummy	1918		316		954		194	
Male		0.522		0.511		0.532		0.460
Female		0.478		0.489		0.468		0.540
The birth order dummies of children	1918		316		954		194	
First child		0.435		0.781		0.424		0.776
Second child		0.342		0.188		0.332		0.237
Third child		0.133		0.028		0.140		0.089
Fourth child		0.089		0.003		0.104		0.003

*Note*. DPT = diphtheria, pertussis, and tetanus; Turkey Demographic and Health Survey.

## 4 RESULTS AND DISCUSSION

### 4.1 First stage estimates

The results of the DDD analysis are presented in Table 3. First, all DDD coefficients are positive and statistically significant as expected. More importantly, the F‐statistics test of the joint significance of the triple interaction term (the instrument) is more than 10 for almost all specifications. This indicates that the instrument is strong (Staiger & Stock, 1997). Also, the instrument has a strongly positive effect on education in all specification. The effect is significant not only statistically but also economically. Considering Column 8, every additional 1 Turkish Lira (TL) of public spending per 1,000 children raised primary school completion by 0.3 percentage points. The average increase in public expenditure on education was 40.36 TL. So it can be said that the education reform caused an increase in the probability of completing at least 8 years of education by 12.1 percentage points (i.e., 0.3 multiplied by 40.36). Given that 17% of women attained 8 or more years of education in 2003, this would represent approximately a 70% increase in the share of women who remained in school for at least 8 years. Similarly, an additional TL spent per 1,000 children increases education by 0.011 years (Column 4). As before, the average additional public expenditure on education is 40.36. Therefore, the education reform caused an increase in years of education by about 0.44 years (162 days). The average length of schooling for the young cohorts in 2003 is 4.91 years. The CEL, thus, lead to a 9% increase in the years of education.

**Table 3:** The impact of the Compulsory Education Law on formal schooling‐DDD analysis (first stage of IV regression)

Dependent variable		Years of schooling				Completing 8 years of schooling		
	Column 1	Column 2	Column 3	Column 4	Column 5	Column 6	Column 7	Column 8
treatment*intensity*2008	0.014***	0.009***	0.009***	0.011***	0.003***	0.002***	0.002***	0.003***
	(0.004)	(0.004)	(0.003)	(0.003)	(0.000)	(0.000)	(0.000)	(0.000)
Controls								
Ethnicity	No	Yes	Yes	Yes	No	Yes	Yes	Yes
Rural/urban status during childhood	No	Yes	Yes	Yes	No	Yes	Yes	Yes
A child‐gender dummy	No	No	Yes	Yes	No	No	Yes	Yes
The birth order dummies	No	No	No	Yes	No	No	No	Yes
R‐squared	0.779	0.810	0.810	0.826	0.370	0.436	0.436	0.464
F‐statistics	14.97	7.00	7.24	12.40	44.96	34.84	35.33	39.34
Observations	3,339	3,327	3,327	3,327	3,339	3,327	3,327	3,327

*Note*. Women aged 18–29 in 2003 and 2008 form the sample of analysis. Women aged 18–21 form the treatment group. The intensity is the difference between the 1997 and 1996 government funds distributed for primary school construction at the region of childhood. Robust standard errors in parentheses cluster at the region of the childhood. F‐statistics are the test of the joint significance of the triple interaction term, that is, the instrument (treatment*intensity*2008). The baseline Models 1 and 5 include no control variable. In addition to the controls given in the table, all models include ethnicity, the urban/rural status of the region of residence in childhood (except Models 1 and 5), the region of childhood, year of birth and age of respondent fixed effects, the intensity variable, the interaction of year of birth with gross enrolment rate in the region of childhood, and the interaction of treatment and intensity variables. DDD = difference‐in‐difference‐in‐difference.

* *p* < .1.

** *p* < .05.

*** *p* < .01.

Our analysis compares young mothers between the ages of 18 and 21 in the 2003 and 2008 TDHS with those aged 22–29 in the same surveys. The young mothers in the 2008 TDHS were exposed to the reform. To test the validity of our methodology, we now replace the three‐way interaction terms in Equation (2) with 12 separate dummy variables, one for each year of age. As expected, the estimates of the coefficients for mothers aged 22–29 are close to zero and statistically insignificant for both years of education and primary school completion (see Table 4). In contrast, the coefficients are statistically significant and positive for women aged 18–21. 15We also run a logistic regression to estimate results presented in Table 4, and the marginal effects from the logistic regression shows similar effect of the CEL for each age.


**Table 4:** The impact of CEL on the schooling of each age cohort separately

	Dependent variables	
Age in 2008	Completing 8 years of education	Years of education
18	0.004*** (0.001)	0.027*** (0.008)
19	0.004*** (0.001)	0.029*** (0.005)
20	0.004*** (0.001)	0.024*** (0.006)
21	0.003*** (0.000)	0.009** (0.004)
22	0.000 (0.000)	0.004 (0.004)
23	0.000 (0.001)	−0.003 (0.004)
24	0.000 (0.000)	−0.002 (0.004)
25	−0.000 (0.000)	−0.002 (0.002)
26	0.000 (0.001)	0.001 (0.004)
27	0.000 (0.000)	0.004 (0.003)
28	0.000 (0.000)	0.001 (0.003)
Observations	3,327	3,327

*Note*. This Table shows the impact of CEL on primary school completion rates and single years of education for each age. The estimation sample covers mothers aged 18–29 at the time of the surveys. The interaction term is the interaction of age*intensity*2008 for each age. Robust standard errors are in parenthesis. Standard errors are clustered at the region of childhood. CEL = Compulsory Education Law.

* *p* < .1.

** *p* < .05.

*** *p* < .01.

### 4.2 RF, OLS, and IV estimates

Starting with the RF estimates, the results in Table 5 indicate that as a consequence of the CEL, there is a statistically significant increase in the probability of the third dose of DPT (Hepatitis B) being administered for the cohort affected by the education reform. Turning to the OLS coefficients, an additional year of maternal education is associated with 1.3 and 1.4 percentage point rise in the likelihood of complete immunization status of infants for DPT3 and Hepatitis B3, respectively. Completing 8 years of formal schooling results in an increase in the probability of vaccination of around 5% for DPT3 and 7% for Hepatitis B.

**Table 5:** The causal impact of education on the complete vaccination status of children aged over 6 months

	DPT3	Hepatitis B
Reduced form estimates	0.001** (0.001)	0.002*** (0.001)
OLS estimates		
Years of education	0.013*** (0.004)	0.014*** (0.003)
Completing 8 years of schooling	0.048* (0.025)	0.070*** (0.023)
IV estimates		
Years of education	0.127** (0.054)	0.215*** (0.058)
Completing 8 years of schooling	0.546** (0.216)	0.920*** (0.214)
Observations	3,325	3,326

*Note*. Women aged 18–29 in 2003 and 2008 form the sample of analysis. Women aged 18–21 form the treatment group. The intensity variable is measured as the difference between the 1997 and 1996 government funds distributed for primary school construction at the region of childhood. Robust standard errors in parentheses cluster at the region of childhood. For the analysis of all types of regressions, Model 8 is used. Therefore, all models include ethnicity, the urban/rural status of the place of childhood, region of childhood, year of birth and age of respondent fixed effects, the intensity variable, the interaction of year of birth with the gross enrolment rate in the region of childhood, the interaction of treatment, and intensity variables. DPT = diphtheria, pertussis, and tetanus; IV = instrumental variable; OLS = ordinary least squares.

* *p* < .1.

** *p* < .05.

*** *p* < .01.

The IV estimates similarly indicate a positive and significant causal effect of maternal education on vaccination status. 16Similar to Currie and Moretti (2003), we rerun Model 4 for the sample of first time mothers. The findings are robust to removing mothers with previous children. The results are available upon request. Specifically, an additional year of schooling increases the probability of receiving the third dose of DPT and Hepatitis B by around 13% and 22%, respectively. Completion of 8 years of schooling increases the probability of receiving the third dose of these vaccines by 55% for DPT and by 92% for Hepatitis B. Hence, maternal education has a strongly positive significant effect on their children's vaccination rates. 17In an unreported regression, we add additional controls to test the robustness of the IV estimates. To do this, we add the age dummies of the children, the age at first marriage and birth, husband's and respondent's labor force status, household size, women's attitudes toward gender equality and domestic violence, and the wealth index of the family. Even after controlling for these covariates jointly or separately, the IV estimates are robust and similar to those in Table 5. In addition, both IV and DDD results are robust to estimating the results with a more balanced sample of women in the treatment and control groups (i.e., four young cohorts and four older cohorts).


It can be seen from Table 5 that the IV coefficients are several times larger than the OLS ones. This can be attributed to the following reasons. First, the IV technique addresses the endogeneity of education whereas OLS does not. Second, according to Imbens and Angrist (1994), the IV coefficients capture the Local Average Treatment Effect, which is the marginal effect of education on the dependent variable for those who altered their schooling choice due to the reform. It is assumed that because of higher marginal cost of schooling, those people would choose lower years of schooling in the absence of the reform. In additıon, assuming higher return to schooling for these individuals, the IV estimates will result in larger coefficients. Having said that, we can still obtain reliable coefficients for the causal correlation from maternal education to immunization outcomes for that subpopulation (Imbens & Angrist, 1994).

## 5 CONCLUSIONS

This paper is, to the best of our knowledge, the first study providing evidence as to whether the observed correlation between maternal education and childhood immunization rates implies causation. To do this, we use a natural experiment from Turkey: the adoption of the CEL which led to an exogenous increase in the compulsory schooling from 5 to 8 years for those born after 1986. This, in turn, has led to an increase in spending on the construction of new classrooms and employment of new teachers. Importantly, the additional spending on teaching infrastructure varied substantially across the regions of Turkey. This paper uses the regional variation in the intensity of the CELs application as an instrument for the schooling of young women aged 18–21, in order to investigate the causal relationship between maternal education and the intake of the third (last) dose of the Hepatitis B and DPT. The DDD methodology employed in this study ensures that rather than comparing relatively old and young mothers, we are comparing groups within the same age ranges between two periods, 2003 and 2008. We find that an exogenous rise in maternal education improves the completion of the last dose of Hepatitis B and DPT immunization, even after controlling for the gender and birth order of the child. These results seem to be very robust to changes in the regression definition and controlling for individual and community level variables.

We only consider the effect of maternal education on children's immunization status and disregard the potential effect of fathers' education, for two reasons. First, in Turkey, as in many other socially conservative countries, gender roles are quite clearly defined. Child care is typically the responsibility of the mother. Second, the women affected by the compulsory education reform were aged 18–21 when they participated in the 2008 survey. Given that women usually marry men who are older than them, most of their husbands were not affected by the reform. Indeed, among the women included in the 2008 survey, only 36 have husbands aged 18–21. With so few observations, a meaningful analysis of the effect of paternal age on children's immunization would not be possible. This also means that the effect we capture should be mainly that of the maternal education. Later analyses, with both parents exposed to the reform, might confound the maternal and paternal effects. Similarly, in the future, immunization rates can increase further because of spillover effects from other family members exposed to the reform. Finally, higher education of mothers can have further effects due to assortative mating, whereby educated women tend to have husbands with similar level of education. 18We are grateful to an anonymous referee for suggesting this potential effect. Because this effect can occur even when the husbands are too old to have been exposed to the reform, we are not able to control for it. 19In related work, Özer and Fidrmuc (2018) find that women's education lowers the probability that women experience domestic violence and are less likely to be in a marriage that was arranged without their consent.

